# The Promise of AI for Image-Driven Medicine: Qualitative Interview Study of Radiologists’ and Pathologists’ Perspectives

**DOI:** 10.2196/52514

**Published:** 2024-11-21

**Authors:** Jojanneke Drogt, Megan Milota, Wouter Veldhuis, Shoko Vos, Karin Jongsma

**Affiliations:** 1 University Medical Center Utrecht (UMC Utrecht), Heidelberglaan 100, Utrecht, 3584 CX, Netherlands; 2 Radboud University Medical Center, Nijmegen, Netherlands

**Keywords:** digital medicine, computer vision, medical AI, image-driven specialisms, qualitative interview study, digital health ethics, artificial intelligence, AI, imaging, imaging informatics, radiology, pathology

## Abstract

**Background:**

Image-driven specialisms such as radiology and pathology are at the forefront of medical artificial intelligence (AI) innovation. Many believe that AI will lead to significant shifts in professional roles, so it is vital to investigate how professionals view the pending changes that AI innovation will initiate and incorporate their views in ongoing AI developments.

**Objective:**

Our study aimed to gain insights into the perspectives and wishes of radiologists and pathologists regarding the promise of AI.

**Methods:**

We have conducted the first qualitative interview study investigating the perspectives of both radiologists and pathologists regarding the integration of AI in their fields. The study design is in accordance with the consolidated criteria for reporting qualitative research (COREQ).

**Results:**

In total, 21 participants were interviewed for this study (7 pathologists, 10 radiologists, and 4 computer scientists). The interviews revealed a diverse range of perspectives on the impact of AI. Respondents discussed various task-specific benefits of AI; yet, both pathologists and radiologists agreed that AI had yet to live up to its hype. Overall, our study shows that AI could facilitate welcome changes in the workflows of image-driven professionals and eventually lead to better quality of care. At the same time, these professionals also admitted that many hopes and expectations for AI were unlikely to become a reality in the next decade.

**Conclusions:**

This study points to the importance of maintaining a “healthy skepticism” on the promise of AI in imaging specialisms and argues for more structural and inclusive discussions about whether AI is the right technology to solve current problems encountered in daily clinical practice.

## Introduction

Image-driven specialisms such as radiology and pathology are at the forefront of technological innovation in medicine, and many believe that artificial intelligence (AI) is the next innovation to reshape these fields [1-3]. AI refers to a broad range of machine-based systems designed to influence the environment by producing an output (predictions, recommendations, or decisions) for a given set of objectives [4]. AI is considered promising for image-driven medical fields because the work involves pattern recognition and is often digitalized, meaning rich datasets are available for AI training. Some have already argued that the professional roles of radiologists and pathologists will drastically change due to AI; they will become “information specialists” [5] or “imaging consultants” [6] who seamlessly use AI to help interpret patient data. Jha and Topol [5] even speculate that the fields will most likely merge, leading to “a natural fusion of human talent and artificial intelligence. United, radiologists and pathologists can thrive with the rise of artificial intelligence.”

Despite the great promises for image-driven diagnostics and Dr Geoffrey Hinton’s prediction that radiology as a specialization would now be extinct, the implementation of AI in routine patient care is often lagging [7,8]. One cause is the lingering uncertainty among professionals about the added value for clinical practice. Another contributing factor is the large variance in acceptance and trust of direct and indirect adopters [9]. While fears about an upcoming “AI winter” [10] are likely unfounded, expectations must be tempered to prevent disillusionment. It is therefore relevant to consider “how to actually deploy AI in clinical practice” and investigate whether the high expectations of AI in radiology and pathology require substantial changes in these fields—and in the current implementation approaches used by AI vendors [7].

Empirical studies have investigated how image-driven professionals view AI innovations. For example, professionals in radiology [9,11-14] and pathology [15-17] have a wide range of predominantly positive expectations for AI, yet they remain divided on the roles AI should have in their daily workflows. Studies have also called for a more thorough incorporation of medical professionals’ views in AI design and implementation [12,18]. This paper aims to add to the understanding of image-driven professionals’ views on the future of AI in radiology and pathology by highlighting how their views relate to current discussions on AI. As far as we are aware, this is the first qualitative interview study to combine views from both fields. By doing so, we hope to provide a more comprehensive perspective on AI’s influence on medical imaging. These insights are also intended to help inform the responsible integration of AI in image-driven medicine.

## Methods

### Overview

This study is part of a broader research project focusing on the ethical integration of AI in image-driven medicine, and the main research question is, “how should AI be responsibly integrated and used in image-driven medicine?” In order to answer this question, we use empirical research methods such as qualitative interviews and participant observations [19] to ground and inform our ethical analysis. The interview study design is in accordance with the consolidated criteria for reporting qualitative research (COREQ) [20]. In another publication, we reported the perspectives of pathologists, laboratory technicians, and computer scientists from 2 Dutch hospitals regarding the development and implementation of AI in pathology [15]. For the previous paper, we focused on the perceived roles and responsibilities of AI according to professionals working in pathology. In this paper, we compare and contrast the perspectives of professionals working in 2 departments—radiology and pathology—within 1 Dutch hospital and focus on the perceived promise of AI for image-driven medicine.

### Research Design

To gain insight into the promise of AI for image-driven medicine, we conducted an inductive qualitative analysis of recorded conversations with radiologists, pathologists, and computer scientists [21-24]. The interviews with computer scientists have been used to contextualize and steer the interpretation of our findings.

### Sampling in a High-Resource Context

Radiologists and pathologists working at 1 academic hospital in the Netherlands, the University Medical Center Utrecht (UMCU), were invited to participate in this study via a department-wide call. Potential participants were also directly approached by the research team or a contact person at the department to reach a representative group of professionals. We personally approached radiologists and pathologists who were less involved in AI integration in these fields because we found it important to include their perspectives in the study. Computer scientists working with these departments were also asked to participate to provide additional context. For several reasons, we chose to focus on professionals from 1 innovation-driven medical center. First, these departments are relatively far along in their AI implementation processes compared with other Dutch hospitals. We hypothesized that this would correlate with a greater familiarity with AI, meaning respondents would be more likely to relate their opinions and expectations to practical encounters with AI. Second, we also recognize that context matters for AI integration and that it can be challenging to compare different medical contexts [25]. Focusing on radiologists and pathologists from 1 medical center enabled the comparison of perspectives on AI innovations between the departments, as, in general, the 2 departments function in the same context (eg, same region, managerial structures, and access to high-quality data), and both have access to internal computer science teams to support AI development and use. Nevertheless, we also recognized that conducting the study at 1 medical center would present a practical challenge. As there were a fixed number of radiologists and pathologists working at these sites, and we were dependent on their willingness to participate, the number of respondents for this study was finite. As our primary aim was to conduct a comprehensive exploration of perspectives on the promise of AI for image-driven medicine, we have focused on including a range of perspectives present in the departments to ensure broad representation instead of purely focusing on the sample size. This means we have taken meaning saturation into account in the analysis of the data to ensure that the quality of data is high and that the elicited views are representative of the perspectives present in the departments (except perhaps for those respondents who remained unwilling to discuss the potential of AI; see the Discussion section), but we mainly reflected on the information power in our in-depth interviews [26,27].

### Data Collection and Analysis

Interviews were conducted between June 2020 and December 2021. Because of the pandemic, many of the conversations took place via the telephone; JD and MM conducted interviews individually and as a team. A semistructured topic list was used to guide the conversations (see Table 1 for sample questions). The recorded interviews were transcribed verbatim by a professional transcription service and checked for reliability by JD. The transcripts were then coded for confidentiality, and identifying information was removed. The interviews were conducted in Dutch and translated to English by JD and MM.

**Table 1.** Sample interview questions of semistructured topic list.^,^

Designators		Questions
**A. General questions about respondent’s background**		
	A1	How long have you worked as a radiologist/pathologist?
	A2	Why did you choose to specialize in the field?
	A3	What technology developments did you encounter during the time you have worked as a radiologist/pathologist?
**B. Question(s) on conceptualization of AI** ^a^		
	B1	In your view, how would you define AI?
**C. Questions about respondent’s thoughts and opinions about AI:** ***What is the perceived effect of the introduction of AI in relation to ideas about professional identity and expertise?***		
	C1	In general, what do you think AI could mean for radiology/pathology?
	C2	To which extent are you involved in AI integration in your field?
	C3	What kinds of AI applications would be most helpful or useful to you?
	C4	In what ways do you think AI might impact your decision-making process?
	C5	What (new) skill(s) or knowledge do you foresee yourself needing if AI becomes more prevalent?
	C6	In the next 10 years, how do you expect AI to impact radiology/pathology?
**D. Questions about desirable ethical guidance for AI in image-driven medicine**		
	D1	What ethical issues do you foresee with the increased use of AI in your work?
	D2	Do you think special guidelines should be established for using AI?If so, what kinds of issues should be addressed?
**E. Exit questions**		
	E1	Do you have any other thoughts or opinions about the use of AI in your department that you’d like to share with me?
	E2	Is there anything you think we missed? Is there an important question I forgot to ask?

a AI: artificial intelligence.

The data selection and analysis occurred inductively and iteratively [28] using constant comparison [29]. The software program NVivo (version 12; Lumivero) supported the data analysis. JD and MM read individual interview transcripts and independently identified conversation fragments or units of meaning [21-24] they considered relevant to the research question; they met regularly to compare their observations. They used the code tree from the analysis for the earlier publication [15] as a baseline, adapted the code tree to fit the new dataset, and supplemented it with new descriptive categories. JD and MM then sampled and independently coded 4 interviews, compared the results, and refined the code tree. JD then coded the remaining transcripts, adjusting the code tree when necessary. Finally, MM and JD performed an intercoder reliability check by recoding 2 interviews (1 pathologist and 1 radiologist) and comparing their results. Meaning saturation and information power were taken into account throughout this process [27,30]. During the analysis, JD, MM, and KJ kept track of new AI developments in radiology and pathology; in consultation with WV and SV, we evaluated the relevance of the data to current situations on the work floor and included current literature in the discussion.

### Data Statement

The data have been presented by means of in-text illustrative quotes, carefully selected to represent the arguments presented in the interviews and do justice to the variety of perspectives captured in the interviews. We have considered whether the quotes could be understood without the context in which they were originally uttered. The complete datasets are not publicly available because privacy of individual participants could be compromised. The individual privacy of the participants was particularly important as their statements included political opinions and philosophical beliefs regarding the ways in which AI should be adopted. These are deemed sensitive and, therefore, fall under the protection of the General Data Protection Regulation (GDPR: article 9).

### Ethical Considerations

This study constitutes part of the Responsible Artificial Intelligence in clinical DecisIOn making (RAIDIO) study. Ethical approval for the RAIDIO study was obtained from the Medical Research Ethics Committee of the UMCU (WAG/mb/20/014090). The Medical Research Ethics Committee determined that this study was exempt from the Medical Research Involving Human Subjects Act. Written or oral informed consent was obtained from all participating respondents. Data were deidentified through pseudonymization and stored in a protected digital environment of the UMCU. Participants of this study did not receive financial compensation.

## Results

**Figure 1.**
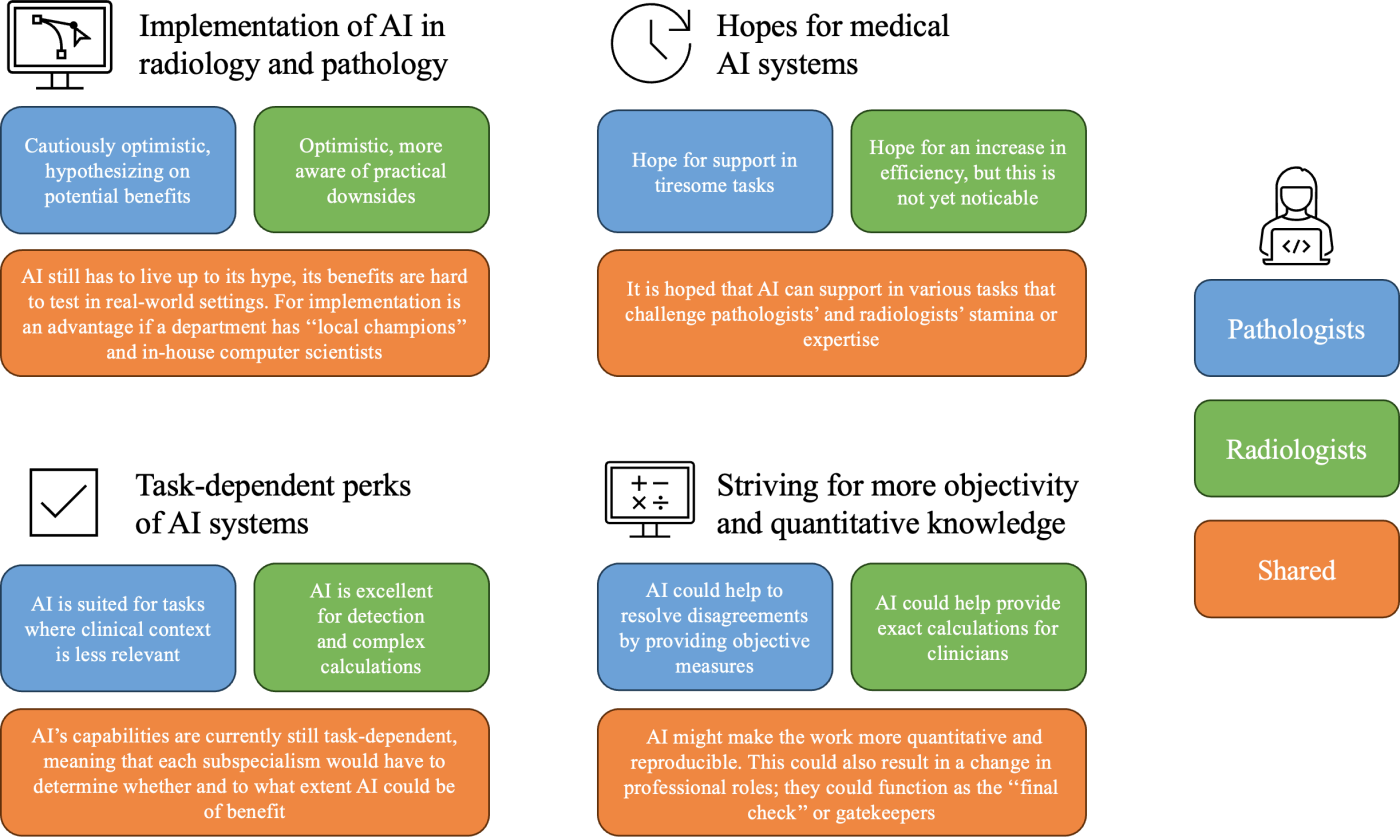
General overview of respondents’ perspectives. AI: artificial intelligence.

In total, 21 participants (7 pathologists, 10 radiologists, and 4 computer scientists) agreed to be interviewed, provided written or oral informed consent, and were included in this study. The following sections present how participants perceived current AI developments and AI’s promise for image-driven medicine; we pay special attention to similarities and differences between radiologists and pathologists (for an overview of respondents’ perspectives, see Figure 1).

### Implementing AI in Radiology and Pathology

Respondents from both fields considered AI a novel technology. The extent to which participants could elaborate on technical or development issues of AI in their respective fields depended on their familiarity and previous experiences with these technologies. Nevertheless, it was striking that *all* respondents could refer to a landmark AI system as a point of reference; for the pathologists, this was a mitosis counting algorithm, and for the radiologists, it was a pulmonary embolism detection algorithm. To some extent, these 2 systems shaped the ways respondents envisioned future AI integrations in their departments. Because of the initial success of the mitosis counting algorithm, pathologists were cautiously optimistic about AI in their field. Many expected that other “simple” tasks could be supported or performed by an AI system, and some considered it a matter of time before applications for more complex tasks would be developed. Most radiologists were also optimistic about AI systems, but multiple respondents referred to minor flaws in the pulmonary embolism detection algorithm when considering possible future AI applications. For example, although the accuracy of the detection tool was very high, they still had to actively verify the algorithm’s outcomes. Some also considered the notifications of possible pulmonary embolisms a disturbance to their workflow.

Both pathologists and radiologists, independent of their respective familiarity with AI, admitted that medical AI in practice had yet to live up to its hype. Besides the fact that it is technically complicated to integrate multiple AI systems into the workflow of professionals, another roadblock respondents mentioned was the difficulty in getting an AI system tested in real-world settings. As a pathologist (P7) described:

*testing [the AI system] in the real world is something that is not standard practice, and you encounter all kinds of problems when you try to do it (…) [AI] functions suboptimally when employed outside the research setting.*

Respondents mentioned that they were lucky to have computer scientists working in or with their departments who were available to help problem solve when integrating new AI systems into their daily practice. Respondents were aware that the close collaboration with data scientists made their departments unique. They considered their departments a “frontrunner” (P7) and example for other hospitals in regard to AI implementation. Both departments also had “local champions,” pathologists, or radiologists who were exceptionally knowledgeable about AI and could “speak the language” of medical specialists and computer scientists. These “local champions” helped accelerate the adoption of AI. As a radiologist (R8) described, AI implementation is site-specific:

*In our hospital, some people are very invested in this topic. We probably encounter more changes than other hospitals. It might go relatively quickly at our department.*

For these reasons, the pathology and radiology departments of the UMCU may have a head start in working toward (more elaborate) AI implementation and are at a point in which they must decide how to proceed. In the following sections, we describe how pathologists and radiologists articulated the changes they were experiencing and how they envisioned AI’s impact on the future of their specialties.

### Hopes for Medical AI

Most radiologists and pathologists in this study argued that AI could help improve medical care by supporting tasks that challenged their stamina or expertise. This is illustrated by an overview of AI systems that were present in the departments and which were referred to in the interviews (Table 2). These systems fall into one of three categories: (1) fixes for tiresome or time-consuming tasks, (2) support in cases where context and classification are challenging according to current standards, and (3) prognosis and therapeutic response (prediction) generators.

**Table 2.** Overview of AI^a^ systems at the radiology and pathology departments^b^.

Field	Specific medical task	Potential role of AI algorithms	Relevant to which (sub)specialisms	Benefit mentioned by pathologists and radiologists	Level of risk involved	Stage of development
Pathology	Determining the aggressiveness of a tumor	Counting the number of mitoses on a digital slideCalculating the percentage of Ki67 positive tumor cells (proliferation index)	Pathology, medical oncology, pulmonary medicine, endocrinology, etc	Tiresome task, less subjectivity	Low—can be checked manually	Implemented
Pathology	Grading cancer; for instance, grading of breast and prostate cancer	For example, Bloom and Richardson grading score for breast cancer	Pathology, medical oncology, urology, etc	Tiresome task, less subjectivity	Low—can be checked manually	In development
Pathology	Analyzing the inflammatory response	Identifying as well as quantitative measurement of number and distribution of immune cells, for example, in/around tumors	Pathology, medical oncology, internal medicine, etc	Tiresome task, less subjectivity	Low—can be checked manually	In development
Pathology	Deciding whether to proceed to surgical (vs endoscopic) resection in case of early colon carcinoma	Analysis of tumor characteristics related to the patient’s chance of developing (future) metastases of colon carcinoma	Pathology, gastroenterology, and surgery	Less invasive treatment for the patient, personalized medicine	High—not easy to check	Research phase
Pathology	Determining prognosis and treatment options for patients with cancer	Analysis of prognostic and (treatment) predictive tumor characteristics	Pathology, medical oncology, pulmonary medicine, etc	Less subjectivity, personalized medicine	High—not easy to check	Research phase
Pathology	Analyzing naevi and other melanocytic lesions on signs of malignancy	Analysis of characteristics associated with malignancy, providing reasons for why the sample is malignant or not, or calculate the risk of malignancy	Pathology, dermatology, and medical oncology	Less uncertainty about the diagnosis, learning from the algorithm	High—not easy to check	Research phase
Pathology	Generating the pathology report	Generating an initial pathology report for a pathologist to check	Pathology	Tiresome task, less variation in reporting style between pathologists	Low—can be checked and changed manually	Research phase
Pathology	Checking images on possible metastases in lymph nodes	Initial screening of lymph nodes on possible metastases	Pathology	Tiresome task	Low—can be checked	Research phase
Radiology	Confirm/rule out pulmonary embolism	Detect/rule out suspected pulmonary embolisms on dedicated CTPA^c^ scans	Radiology, internal medicine, and cardiology	Faster diagnostic process in case of confirmed high accuracy	Low in terms of patient risk: dedicated CTPA are always checked for the primary rule out PE^d^ question. But high level of trust required for benefit to be realized	Implemented
Radiology	Detect incidental pulmonary embolism	Detect incidental pulmonary embolism on CT^e^ scans made for other indications	Radiology, oncology, trauma, internal medicine, cardiology, etc	Earlier detection of unsuspected PE in nonprioritized scans + increased detection rate of unsuspected incidental PE in general	Low—is primarily added value of current standard of care	Implemented
Radiology	Measure prostate in 3D and manually calculate both corresponding prostate volume estimate and its ratio with the plasma PSA^f^ value to determine PSA density correlated with risk of prostate cancer being present	Measure actual prostate volume in 3D (+ provide PSA density)	Radiology, urology, oncology, and radiotherapy	Tedious and repetitive task	Low—volume calculation performed by AI and the corresponding segmentation on which the calculation depends is easily visually checked by the radiologist	Implemented
Radiology	Determine age of pediatric patient on the basis of hand x-ray	Independently perform the bone-age determination	Radiologists and pediatricians	Fully automated procedure	High—bone-age independently determined by AI—with only a visual check of the correctness of joint segmentation by the radiologist	Implemented
Radiology	Detecting and measuring lung nodules	Detecting lung nodules including quantitative 3D volumetry	Radiologists, pulmonologists, oncologists, etc	Tedious, repetitive task, and possible reduction in number of missed nodules	Intermediate—aids in detection, volume calculation more quantitative than radiologist, but correlation with prior scans (crucial for determining growths over time) still lacking in reliability and intuitiveness	Implemented
Radiology	Detecting cervical spine fractures	Detecting fractures in cervical vertebral bodies on CT scans that include the neck	Radiologists, trauma surgeons, orthopedic surgeons	Quicker diagnostic process—theoretically reduced number of missed fractures	Low—always checked	Implemented
Radiology	Quantify cerebral white matter disease	Quantifying the volume of white matter lesions on MRI^g^ of the brain	Radiologists, neurologists	More quantitative and more reproducible measurements, including individualized comparison to reference standard	Low—correctness is easily and reliably visually verified	Research phase
Radiology	Working toward body composition–derived prognostication and personalized treatment	Quantifying the volume of multiple different muscle groups and of subcutaneous and visceral fat	Radiologists, any clinical profession ordering CT scans containing the abdomen	Impossible to perform by radiologists (far too time-consuming, would be hours of work per scan)	Low—with respect to the correctness of segmentations (important to understand that the prognostic application is not part of the AI output)	Actively used in research setting in clinical trials
Radiology	Working toward body composition for personalized drug dosing; from contrast agents to chemotherapeutics	Quantifying the volume of multiple different muscle groups and of subcutaneous and visceral fat	Radiologists, any clinical profession ordering CT scans containing the abdomen	Impossible to perform by radiologists (far too time-consuming, would be hours of work per scan)	High—while the segmentation is reliably verified, subsequent drug dose calculations require extensive validation	In development
Radiology	Segmenting the liver: both the organ and its internal liver segments for subsequent clinical and treatment calculation that depend on liver/segment volumetry	Segmentation of liver and liver segments	Radiologists, interventional radiologists, nuclear medicine, HPB^h^ surgery, oncology	Time-consuming, tedious, task-automated, and made more reproducible	Intermediate—easily, visually checked, still requires some manual corrections	In development
Radiology	Working toward body composition for creatinine clearance calculations that are both more personalized and do not require 24-hour urine samples	Quantifying the volume of multiple different muscle groups and of subcutaneous and visceral fat	Radiologists, any clinical profession ordering CT scans containing the abdomen	Impossible to perform by radiologists (far too time-consuming, would be hours of work per scan)	High—while the segmentation is reliably verified, subsequent drug dose calculations require extensive validation	In development
Radiology	Deciding whether a patient undergoing a breast MRI for detection of breast cancer needs additional imaging or can exit the scanner	Triage of patients with and without possible breast cancer based upon the initial phases of enhancement directly after contrast injection	Radiologist, MRI technologist	Reducing examination time from ±25 minutes to ±5 minutes	High—not easy to check	In development

a AI: artificial intelligence.

b Check for completeness by radiologists and pathologists working at the department.

c CTPA: computed tomography pulmonary angiogram.

d PE: pulmonary embolism.

e CT: computed tomographic.

f PSA: prostate-specific antigen density.

g MRI: magnetic resonance imaging.

h HPB: hepato-pancreato-biliary surgery.

The hope that AI can support or take over time-consuming tasks was especially prominent in the interviews. Many respondents were concerned about the increased work pressure, as clinicians often depend on radiologists’ and pathologists’ knowledge to diagnose and treat patients. One respondent (R7) described their relationship with clinicians as follows:

*There is almost no patient who (…) is treated without scans. We are constantly discussing patients with [other] medical disciplines. These meetings cost a terrible amount of time. Everyone wants you to be at their beck and call*.

Respondents appreciated that their fields were seen as essential to the medical system and that their perspectives were valued. Still, many worried about the workload and the limited time to assess cases, write reports, and prepare for multidisciplinary meetings. Pathologists and radiologists were optimistic about AI’s future role in time-consuming activities, such as tissue or tumor segmentations, calculation of abnormalities such as deviations in heart function, and detection of the evolution of brain metastases or the presence of tumor cells in lymph nodes. In other words, by supporting these kinds of tasks, AI could help them refocus on the more “enjoyable” aspects of the job, such as diagnosing complex disease patterns. As a radiologist (R9) mentioned:

*It would be fantastic if part of our routine work (…) could be taken over. I hope that this will be possible in the future, so we radiologists can again focus on the fun things*.

Nevertheless, some also worried that AI applications would not increase efficiency and might even cost them extra time; as one respondent stated:

*In the meantime, the amount of scans increases, and I’ll also have to manage the AI. That’s something to think about. Eventually, we’ll just be doing our jobs. But hopefully, the quality will become a little bit better*. [R10]

Respondents thus speculated about the impact of AI on the work pressure they experienced. This was particularly apparent for radiologists who noticed that the pulmonary embolism algorithm resulted in a quicker diagnostic process. Using the algorithm also meant that they had to recheck a patient’s images when notified of a possible embolism.

### Task-Dependent Perks

Notably, radiologists and pathologists put their hopes of AI in perspective by remarking that it is task-dependent, meaning that each subspecialism would have to determine whether and to what extent AI could benefit their work. Besides naming technical hurdles, many respondents noted that the amount of input needed to make a diagnosis or prognosis would likely determine whether AI would be suitable for their diagnostic process. For instance, respondents did not expect AI to be able to make complex integrations between different sources of knowledge or to prioritize information. As R7 argued, work in the field requires:

*Integrating everything you’ve learned in your medical education and training as a radiologist, (...) I sometimes wonder how AI could help me with this. I think it will be useless on this front. AI (...) doesn’t know how to search through old reports and gather the relevant information for my scans. I think this will remain—as I currently see it—a skill particular to medical experts*.

As the quote illustrates, respondents questioned whether AI would be useful when a radiologist or a pathologist had to determine which information was relevant for interpreting a medical image, a common practice in all but the most straightforward cases. Although some respondents mentioned that AI could provide a differential diagnosis based on context-related information (eg, age, gender, or laboratory results), many doubted whether AI could prioritize or “weigh” this information in the same way they did. As one pathologist (P1) commented:

*I think that context certainly matters. AI could go wrong because it insufficiently weighs the context. (...) Plus I also think a lot of histological images look similar. But the clinical context of one patient may be very different from another patient and will result in another diagnosis, even when the images look completely the same. (...) One histological image could indicate 20 different clinical diagnoses, especially if you’re looking at images of inflammatory disease. This won’t be easy for AI*.

Radiologists and pathologists often viewed “real interpretation” (R3) as something exceeding the capabilities of AI. They mentioned that AI could be good at detecting certain things (such as lung nodules or other conspicuous manifestations of cancer) and might even gain an “associative capacity” (R1) similar to their own, but that it would increase their workload if adopted in areas where they did not need it. Some respondents had not ruled out the possibility of AI becoming better at specific, well-defined “expert tasks” and found it an exciting thought that AI could become more competent than humans in the interpretation process. Nevertheless, respondents also stated that it would be hard for an algorithm to learn to independently evaluate pathological processes with respect to the clinical context, making many of the potential uses for AI more speculatory than an inevitability.

### Striving for More Objectivity and Quantitative Knowledge

Although there were varying views on the tasks best suited for AI, many radiologists and pathologists stated that AI could improve the quality of their work. This was often mentioned with the expectation that AI might make the work less “subjective” and more quantitative and reproducible. For instance, pathologists, in particular, talked about the possibility of AI (sometimes referred to as “the computer”) helping resolve disagreements in their fields by offering an additional, objective interpretation of medical images. As one pathologist (P5) stated:

*If you have a tumor cell with a nucleus that is a little bit enlarged, one pathologist could say something like “alright, it’s probably reactive,” and the other pathologist says “oh no, it is malignant.” But a computer could precisely measure the nucleus and determine “Okay, there is a lot of chromatin, it is irregular, this is the intensity of the chromatin.” These are all objective measures by which you could say whether it is benign, malignant or reactive*.

Multiple respondents also discussed a potential beneficial characteristic of AI, namely, that it could keep track of minute details in medical images. It might, therefore, become better than humans at recalling and comparing image characteristics.

AI was also described as a tool to help radiologists and pathologists better understand the data by quantitively measuring multiple aspects of medical conditions. AI systems have already been designed to compute a patient’s fat and muscle mass, the amount of white matter in the brain, the volumes of various parts of the brain, and the histological parameters of a tumor. Some participants mentioned that these AI applications mirror a broader trend in radiology and pathology to approach medical findings in a more quantitative manner. A radiologist (R8) described this as an ongoing shift in the way medical images are used in the field, adding: “It’s not just about the interpretation of images, but also the generation of scores and the production of numbers*.*” Some radiologists also referred to clinicians’ wishes that they provide exact calculations. One radiologist (R5) even called this “the ultimate goal” of their specialism: to precisely identify a patient’s condition for the clinician. Many respondents mentioned that such quantitative measures might also lead to more reliable and precise prognoses by giving the clinician more relevant information to determine a patient’s treatment.

Both pathologists and radiologists also reflected broadly on how AI might change how they form medical judgments. While some radiologists imagined that they would become “data specialists” (R8) or “translators” (R4) who would mainly check the algorithms’ reports, most radiologists and pathologists were inclined to describe themselves as the “final check” or gatekeeper. In other words, they were comfortable letting AI do some of the primary work but wanted the medical specialist to make the final judgment and bear the responsibility for the diagnosis. We observed some slight differences between radiologists and pathologists regarding the role of AI in their specialism. Radiologists seemed more inclined to describe specialism-wide changes initiated by AI and viewed AI as a more significant force that could become an integral part of their specialization. Pathologists primarily focused on AI as an innovation from which they could learn. Respondents from both fields indicated that they were unsure of the ultimate impact AI would have on their specialisms, and when asked for their expectations for the coming 10 years, most replied that they did not expect any fundamental changes to their professional roles or responsibilities.

## Discussion

### Principal Findings

This qualitative interview study investigated how professionals from the 2 most image-driven medical specialisms perceive the promise of AI for their respective fields. Overall, our analysis shows that pathologists and radiologists have comparable views on AI’s possible benefits and drawbacks. Differences between radiologists’ and pathologists’ perspectives were mostly a level of degree; for instance, the use of AI for quantification purposes seems to be somewhat more pronounced in radiology. One reason for this discrepancy might be that the radiology department in our study currently has more experience with implementing AI systems in practice.

The radiologists and pathologists in our study echoed some of the findings of earlier empirical studies concerning the potential of AI in these fields. Respondents in this study also argued that AI could provide them with quantitative data [11] and were interested in AI systems that could perform simple yet time-consuming and repetitive tasks [12]. However, they also worried that AI could result in more work [11] and hypothesized that AI would be less suitable for complex, variable, or intellectually challenging tasks [31]. Respondents from both disciplines (irrespective of their experience with AI) also cautioned about overstating the benefits of AI and tried to shift the focus to task-specific advantages. This resembles the results of the study by Hendrix et al [14], where respondents emphasized that AI-based decision support is contingent on its specific features and functionality. In our study, most respondents had a positive yet realistic view of AI, keeping in mind the current limits of AI and roadblocks for successful implementation.

As our findings reflect the unique combination of pathologists’ and radiologists’ perspectives from a technologically innovative academic medical center, the interview data can indicate how to proceed with the implementation of AI. In the following sections, we discuss the implications of our findings in relation to broader questions about AI integration within image-driven medicine.

### Will AI Reduce or Increase the Workload of Image-Driven Professionals?

Radiologists and pathologists in this study often mentioned that their workload had expanded over the last decades and that they increasingly participated in multidisciplinary meetings. Therefore, many respondents expressed the hope that AI would help them tackle their demanding workloads. This is consistent with other studies and literature, which point to the possibility of designing AI for the most tiresome and repetitive tasks in radiology and pathology [32-35]. Both radiologists and pathologists in this study mentioned that AI had already been developed for several time-consuming tasks in their departments, and some also hoped that AI would someday help them write their reports.

At the same time, many respondents questioned AI’s ability to contribute to increased efficiency. Many studies confirm that AI should not be considered an augmentation or support tool, not a direct replacement for pathologists or radiologists [36,37]. AI involvement would also result in new tasks for professionals, such as validating AI systems and checking outcomes. Professionals would also have to become more skilled in dealing with AI in their daily work. The amount of extra effort it costs to work with AI highly depends on the specific task and the trust radiologists and pathologists have in the algorithm’s functioning. This was illustrated by respondents’ emphasis that they wanted to remain involved in the final medical conclusion. A similar argument can be found, for example, in the study by Ranjan et al [37]. Literature on the successful adoption of AI in clinical workflows often stresses that physicians should have epistemic trust in AI functioning, adding that many open questions still exist on the level of control physicians should have over AI [38] and which kinds of outcomes physicians should trust [39,40].

The results of this study have highlighted the dichotomous role AI could play in the high workload of professionals working in image-driven fields; they also point to the importance of contemplating the amount of work AI could and should impose. Although AI could create welcome changes in the workflows of these professionals, it also has the potential to become another technology for them to manage and may not always be a legitimate aid to their already busy schedules.

### What Will AI Mean for the Future of Radiology and Pathology?

Many authors contend that AI could lead to significant changes in the professional roles of radiologists and pathologists [6,41-43], and some have even argued that the fields will eventually merge to become the “information specialists” of the medical system [5]. In this study, participants shared the belief that AI could greatly impact how they perform their work and could change their professional roles. At the same time, they emphasized that many of these changes were speculative and unlikely to occur soon.

Because of the speculative nature of the grander promises of AI, Saboury et al [44] argue that “it is critical to improve our understanding of the pitfalls of deep learning and maintain a healthy and constructive skepticism as we explore the tremendous potential of the technology.” Karhade and Schwab [45] also state that this kind of ‘‘healthy skepticism’’—along with engagement and collaboration with technical experts—can support “the development of AI systems that complement and expand our abilities to diagnose, predict and operate,” help sustain informed dialogue, and ask the right questions concerning the use of AI in clinical practice. Therefore, it may be essential to focus on the actual impact AI can have on radiology and pathology and maintain a skeptical attitude in order to ultimately maximize the advantages of AI. For now, this could also mean focusing on AI’s task- and specialism-dependent benefits rather than its broader potential for integrating multiple medical specialisms—even though bridging disciplinary boundaries between radiology, pathology, and other medical fields may eventually benefit the quality of care [46,47].

### How Can We Incorporate Critical Voices in AI Innovation?

There is currently a push toward AI in image-driven diagnostics, illustrated by assertions such as “radiologists who use AI will replace radiologists who don’t.” [48,49] Yet, the question is, who are the radiologists (and pathologists) who do not want to use AI? Who is going to be replaced? The positive voices about AI still outweigh the more critical voices in existing qualitative interview studies [50,51], and it is hard to find medical professionals who contest the possible advantages of using AI in image-driven medicine. Although those who refuse to work with AI altogether may be a relatively small group, we noticed in our recruitment process that professionals who were less convinced of the benefits of AI or were working in subspecialisms less suited to AI were more reluctant to participate in our study than individuals who were already involved in the validation and implementation of AI [9]. We successfully recruited some individuals with skeptical views but were unable to include those few radiologists and pathologists who remained unwilling to consider the potential of AI for radiology and pathology.

While not everyone has to participate in the debate about medical AI, it is important to be aware of the possibility of perpetuating existing bias in empirical studies about AI. Concerns about the issues that could arise by using AI (such as deskilling and the effects of changing practice patterns on AI) [52] persist; we, therefore, urge radiology and pathology departments to create ways to include critical voices in the development of AI in their fields. Accelerating AI integration could force some professionals to use it even when they believe their field “is not ready for AI” [53]. Ideally, consideration should be paid to how *all* users respond to and can accept the involvement of AI in their workflows. As Krupinski [54] formulates:

*Technology development and deployment are critical to improve patient care, health outcomes, and the efficacy and efficiency with which our health care systems achieve these goals, but it cannot take place without considering how it will be accepted and integrated in routine daily use by all stakeholders.*

Although there may be practical roadblocks to ensuring all voices are represented, inclusive communication will help ensure that more specialists are familiar with specific AI systems; this will also ease the transition to using AI in their workflows. A broad representation of perspectives could also benefit developers by supporting them in detecting blind spots in the design and implementation of medical AI and might facilitate trust in the development process.

### What Could Future Research on AI in Image-Driven Specialisms Focus on?

Besides the importance of maintaining healthy skepticism and focusing on the inclusion of critical voices, this study offers additional recommendations for future research. Future research could, for instance, repeat this study when (both) departments are further along in integrating AI into their workflows. Our study was limited in the sense that, although we selected departments that were relatively far along in implementing AI, the integration of AI in health care is generally still in its early phases. This meant that some questions were answered hypothetically. We expect that perspectives will become more concrete when AI becomes more thoroughly implemented into these specialisms. Another consideration for future research is that it is unclear whether the perspectives mentioned here would also apply to the implementation of AI in low-resource settings. We consciously focused on the integration of AI in 2 high-resource departments, which made the perspectives on AI between these departments more comparable and likely also with other high-resource settings. Yet, it is essential to state that the results of this study should not be taken at face value for low-resource deployment environments [25]. Further research is necessary to determine the extent to which the perspectives presented here are also mirrored in low-resource sites.

## Abbreviations

AI: artificial intelligence
COREQ: consolidated criteria for reporting qualitative research
RAIDIO: Responsible Artificial Intelligence in Clinical DecisIOn making
UMCU: University Medical Center Utrecht
